# Supplementary material for: Genetic Analysis of Novel Behaviour Traits in Pigs Derived from Social Network Analysis
Source: Genes (Basel). 2022 Mar 23;13(4):561. doi: 10.3390/genes13040561 (PMC9027576; doi:10.3390/genes13040561)
Supplement: Supplementary file 1 [file genes-13-00561-s001.zip › genes-1646987-supplementary.pdf]

Table S1. Spearman rank correlations (confidence interval) among the phenotypic values of the social network traits of aggressive behaviour.

Trait	Closeness centrality	Degree centrality	Eigenvector centrality	Clustering coefficient
Betweenness centrality	0.62 (0.58, 0.66)	0.80 (0.78, 0.83)	0.60 (0.55, 0.63)	-0.55 (-0.60, -0.51)
Closeness centrality		0.85 (0.83, 0.87)	0.53 (0.48, 0.57)	-0.05 (-0.10, 0.01)
Degree centrality			0.66 (0.62, 0.69)	-0.14 (-0.20, -0.09)
Eigenvector centrality				-0.10 (-0.14, -0.03)

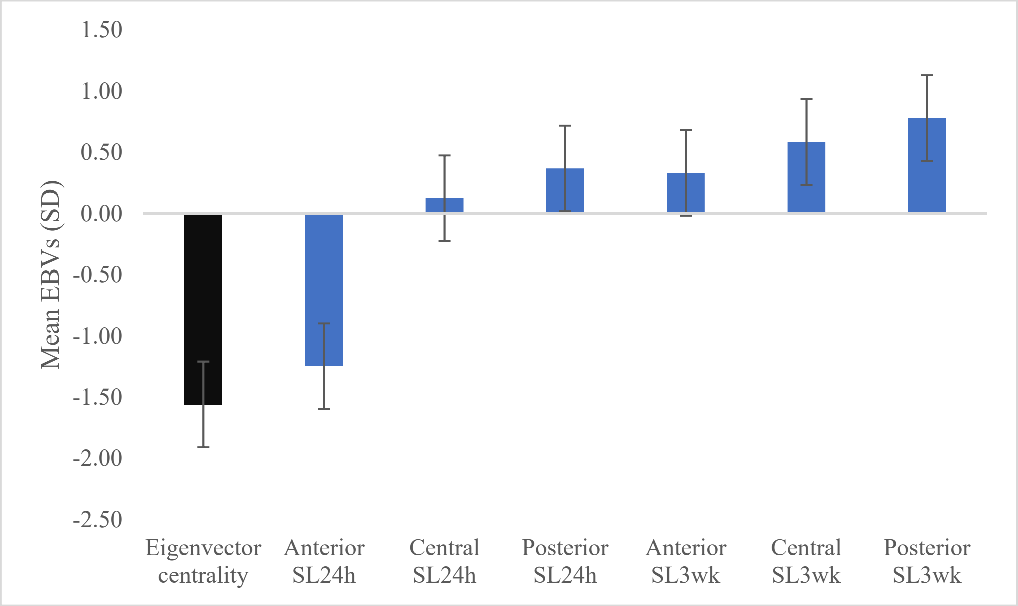

(a)

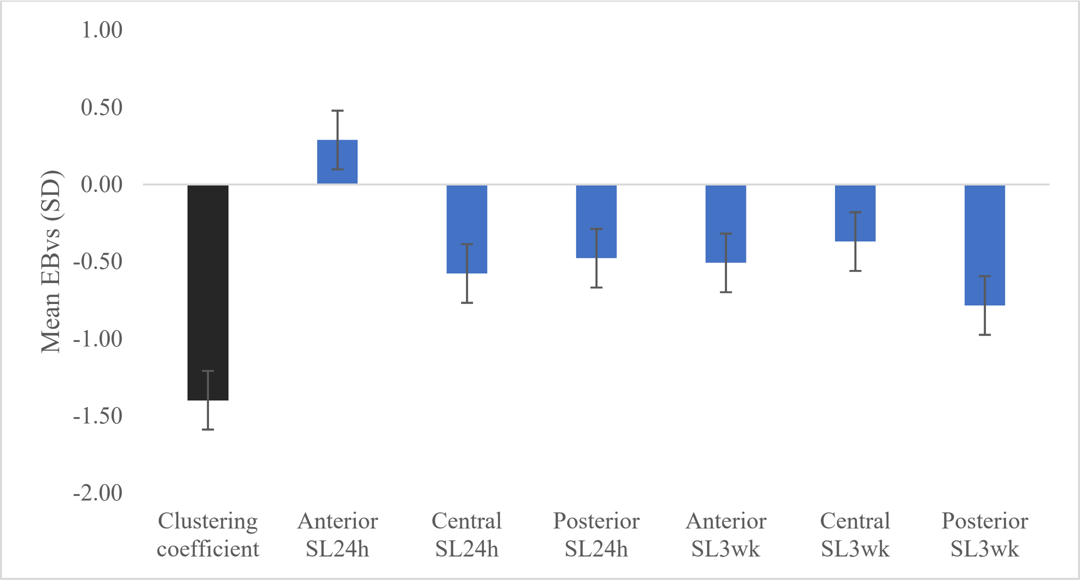

(b)

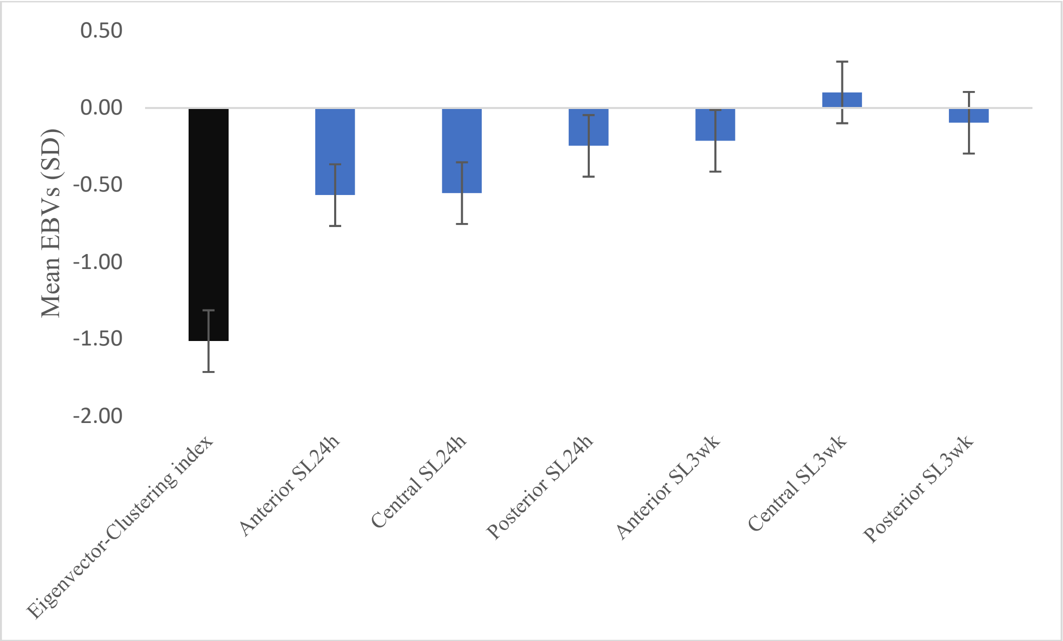

(c)

Figure S1. Mean estimated breeding values (EBVs) of skin lesions traits of pigs with the pen level EBVs in the lowest 20% for eigenvector centrality (a), clustering coefficient (b) and Eigenvector-clustering Index (c). The trait that selection was based on is shaded black.
